# Protective effects of acacetin isolated from Z*iziphora clinopodioides* Lam. (*Xintahua*) on neonatal rat cardiomyocytes

**DOI:** 10.1186/s13020-014-0028-3

**Published:** 2014-12-17

**Authors:** Wei-Jun Yang, Chong Liu, Zheng-Yi Gu, Xing-Yue Zhang, Bo Cheng, Yan Mao, Gui-Peng Xue

**Affiliations:** Key Laboratory of Xinjiang Uighur Medicine, Xinjiang Institute of Materia Medica, Xinhua South Road 140, Urumqi, 830004 China; Institute of Metaria Medica, Zhejiang Academy of Medical Sciences, Hangzhou, 310013 China

**Keywords:** Acacetin, Ziziphora clinopodioides, Neonatal rat cardiomyocytes, Hypoxia/reoxygenation, HPLC

## Abstract

**Background:**

The total flavonoids from ethanol extract of the aerial part of *Ziziphora clinopodioides* Lam. (Lamlaceae) (*Xintahua*) showed protective activities against rat acute myocardial ischemia in rats. This study aims to isolate acacetin, a flavonoid, from the aerial part of *Z. clinopodioides*, to develop an HPLC method for its detection, and to evaluate its protective effects on neonatal rat cardiomyocytes.

**Methods:**

Sephadex LH-20 silicagel and pillar layer chromatography silica gel were applied for the isolation and purification of acacetin and its structure was elucidated on the basis of ^1^H and ^13^C NMR spectroscopy. The content of acacetin in *Z. clinopodioides* collected from three different origins was determined by HPLC. The neonatal rat cardiomyocytes were isolated and cultured *in vitro* to establish a hypoxia/reoxygenation injury model. The viability of cardiomyocytes was measured by the MTT method. Changes of malondialdehyde (MDA) content in the medium were also determined.

**Results:**

The acacetin content in various batches of *Z. clinopodioides* ranged from 45.50 to 47.41 μg/g. Acacetin of 25, 10, 5 μg/mL significantly decreased the MDA content in a model of hypoxia/reoxygenation injury (*P* < 0.001, *P* < 0.001 and *P* = 0.033, respectively).

**Conclusions:**

Acacetin protects neonatal cardiomyocytes from the damage induced by hypoxia/reoxygenation stress through reduction of lipid peroxidation and enhancement of the antioxidant activity.

## Background

*Ziziphora clinopodioides* Lam. (Lamiaceae) (*Xintahua*) is among the commonly used herbal drugs in Traditional Uighur Medicine and Traditional Kazak Medicine, with efficacy for the treatment of hypertension, fever, edema, heart disease, neurasthenic, insomnia, tracheitis, lung abscess and hemorrhoids [1,2]. The pharmacologically active ingredients of *Z. clinopodioides* consist of a large number of iridoids, phenolic and flavonoid compounds, including chrysin 7-*O*-rutinoside, linarin, diosmin, methyl 4-hydroxy-3,5-dimethoxybenzoate, 7-*O*-methylsudachitin (4′,5-dihydroxy-3′,6,7,8-tetramethoxyflavone), thymonin (4′,5,6-trihydroxy-3′,7,8-trimethoxyflavone), caffeic acid and luteolin (3′,4′,5,7-tetrahydroxyflavone) [3–5].

The trichloromethane (CHCl_3_) and ethyl acetate (EtOAc) portions from the ethanol extract of the aerial part of *Z. clinopodioides* showed protective effects on rat acute myocardial ischemia and neonatal rat cardiomyocytes [6]. Detailed analysis indicated that the total flavonoids were the primary contributor to the observed activities [7].

This study aims to isolate acacetin, a flavonoid, from the aerial part of *Z. clinopodioides*, to develop a HPLC method for its detection, and to evaluate its protective effects on neonatal rat cardiomyocytes.

## Methods

### Standards and reagents

MTT was purchased from Amresco LLC (Pennsylvania, USA). Trypsin was from Amresco LLC (Pennsylvania, USA). Streptomycin was produced by North China Pharmaceutical Group Corporation (Shijiazhuang, China). DMEM (high glucose and low glucose) was from Invitrogen Corporation (Carlsbad, USA). Ampicillin sodium for injection was produced from China-promise Pharmaceutical Industry (Shijiazhuang, China). Malondialdehyde (MDA) was provided from the Nanjing Jiancheng Bioengineering Institute (Nanjing, China). Acacetin was prepared in the Key Laboratory of Xinjiang Uighur Medicine (Urumqi, China). HPLC grade methanol and acetonitrile were purchased from Fisher Scientific (New Jersey, USA). Water (0.055 μS/cm) was purified by a Milli-Q system from Millipore (New Jersey, USA). Sephadex LH-20 silicagel was from Amersham Pharmacia Biotech (USA). Pillar layer chromatography silica gel (100-200 mesh) was from Qingdao Marine Chemical Plant (China). All other chemicals were of analytical grade.

### Plant materials

Two batches of *Z. clinopodioides* were collected at the Astronomical Observatory (87°10′40″E, 43°28′14″N, with altitude of 2076 m) and the Chrysanthemum terrace (87°08′38″E, 43°27′14″N, with altitude of 2308 m), South Mountain of Tianshan Mountains in Urumqi, China, in September and August 2010. Another batch was from Xiao Dong Gou (88°07′57″E, 47°56′46″N, with altitude of 970 m) of Altai Mountains in Alaty, China, in August 2010. The plant materials were identified by associate researcher Jiang He (Xinjiang Institute of Materia Medica, Urumqi, China), according to Hudaberbi *et al.* [8], and voucher specimens (no. 100954, 100988, 100990, successively) were deposited in the plant herbarium, Institute of Metaria Medica.

### Apparatus

NMR nuclear magnetic resonance was measured using JEOL ECP-500, INOVA400 and INOVA-600 (Varian, USA). Melting points were measured with a semi-automatic melting point apparatus (Yanagimoto MFG Co., uncorrected. HPLC analysis was performed using a Shimadzu-LC 2010C HPLC (Shimadzu, Japan) system. A BS124S Electronic Balance (Sartorius, Germany) was used for analysis. A DG-5031 ELISA Reader (Nanjing Huadong Electronics Group Medical Equipment Co., Ltd., China) and Shimadzu UV-2501 (Shimadzu, Japan) were used for cardiomyocyte experiments.

### Animals

Neonatal Sprague-Dawley rats (1-3 days old) of either sex were maintained under standard environmental conditions. All animals were purchased from the Experimental Animal Centre of Xinjiang Medical University (Urumqi, China). Certificate Number: SCXK (Xin) 2003-2001. The study protocols were approved by the Ethics Committee on Animal Experiments, Xinjiang Material Medica, China (no.20110515).

### Extraction and isolation

The air-dried aerial portion of *Z. clinopodioides* (10 kg) was extracted with water and then the residue was extracted with methanol (MeOH) under reflux. The methanol extract was suspended in water and then successively extracted with CHCl_3_ and EtOAc. Then, the solution was vacuum-distilled using a rotary vacuum evaporator (Rotavapor R-220; Buchi, Switzerland) to yield the CHCl_3_ fraction (142.5 g) and EtOAc fraction (138.5 g). The EtOAc fraction was purified on silica gel eluted with a gradient of CHCl_3_-MeOH. Eluates were combined according to thin layer chromatography (TLC) behavior using two solvent systems CHCl_3_-MeOH (97:3) to offer compound 1 (320 mg).

### Sample preparation for determination of acacetin

Dried powder (1.0 g) of the aerial portion of *Z. clinopodioides* was refluxed in 30 mL of methanol for 1 h after soaking for 20 min. After extraction, solvent was added to the extraction vessel until the final weight was equal to the starting weight to counter solvent loss. The extract was thoroughly mixed on a vortex mixer, and filtered through a 0.45 μm syringe filter prior to HPLC injection.

### HPLC analysis of acacetin

All experiments were conducted with a Shimadzu-LC 2010C HPLC system. The mobile phase consisted of acetonitrile (A) and water with 1.0% glacial acetic acid (B), with the proportion of A:B held at 37:63. The chromatographic separation was performed using a YMC-Pack ODS-A (4.6 × 250 mm, 5 μm) column with a flow rate of 1.0 mL/min. The column temperature was maintained at 35°C. All analytes were monitored at 326 nm.

### Calibration curves

Stock standard solutions of acacetin were prepared in MeOH and diluted to different concentrations to build calibration curves, *e.g.*, plotting the peak areas versus the concentrations of each analyte.

### Stability test

Sample was analyzed using the developed method to verify the stability of the sample. The stability test was carried out by analyzing the sample at 0, 2, 4, 8, and 24 h. The relative standard deviations (RSDs) of peak areas at different times were calculated.

### Precision test

Intra-day and inter-day variations were used to determine the precision of the developed method. The intra-day precision or inter-day precision was determined by analyzing replicated samples) on 1 day or over 3 consecutive days, respectively.

### Accuracy test

The accuracy of the developed method was evaluated by spike recovery. Acacetin was added into 0.5 g of sample. Then, the mixtures were extracted and analyzed. The spiked recovery was calculated as follows:

$$ Recovery\left(\%\right)=\left[\left( amount\; yield- amount\; original\right) \div amount\; spiked\right]\times 100\% $$

### Cell culture

Primary cultures of neonatal rat cardiomyocytes were prepared from the ventricles of 1 to 3-day-old Sprague-Dawley rats as previously described [9,10]. The cells were pre-plated three times for 30 min in a humidified incubator (95% air/5% CO_2_ at 37°C) in DMEM supplemented with 2 mmol/L L-glutamine, 10% (v/v) foetal calf serum and penicillin/streptomycin (100 U/mL) to minimise fibroblast contamination. Cardiomyocyte-rich cultures (>90%) were plated onto fibronectin-coated 96-well plates, 100 μL per well, at a final density of 1.00 × 10^5^ cells per cm^2^ in supplemented DMEM [11,12].

### Cell viability

The culture solution was discarded after 24 h co-cultivation of myocardial cells and different concentrations of acacetin, and then 180 μL DMEM and 20 μL MTT were added to each well for 4 h cultivation. The supernatant was discarded and 150 μL DMSO was added to each well, mixed evenly, and the absorbance (A) was measured at 570 nm within 10 min using a DG-5031 ELISA Reader [13].

### Model of hypoxia/reoxygenation injury

After cultivation for 72 h, the medium was exchanged with one that was hypoxic (culture medium saturated with high concentrations of N_2_ in advance), and the solution was placed in a hypoxic culture box (99.99% N_2_) for 120 min. Then, the medium was replaced with one saturated with pure O_2_, and the cells were exposed to a normoxic atmosphere containing 95% air and 5% CO_2_ at 37°C (reoxygenation) for 30 min. A thiobarbituric acid (TBA) method was used to determine the content of MDA [6].

### Cell experimental protocol

Experimental doses of acacetin were investigated using a cell viability test. The cells were divided into five experimental groups: group I served as a control (normal cell culture group, incubation for 3 h in the incubator), group II served as the myocardial cell injury control group (hypoxic 2 h, and reoxygenation 1 h), groups III, IV and V were treated with three different doses (25, 10, and 5 μg/mL,respectively) of acacetin (hypoxic 2 h, and reoxygenation 1 h). The inhibition rate was calculated from the absorbance of the medium containing added acacetin over the medium of the control (group I).

### Statistical analysis

The results were reported as the mean ± standard derivation (SD) of at least three measurements. The analysis of MDA data was performed with the SPSS 10.0 statistical package (IBM, USA), while simple linear regression was performed in Excel (Microsoft, Redmond, WA, USA). Results with *P* values less than 0.05 were considered significant.

## Results

### Structural elucidation

Compound 1 was yellow needle-like crystals. ^1^H-NMR(DMSO-*d*_*6*_, 600 M Hz) *δ*: 12.93 (1H, s, 5-OH), 10.87 (1H,br s, 7-OH), 8.05 (2H, d, *J* = 9.0 Hz, H-2′,6′), 7.11 (2H, d, *J* = 8.8 Hz, H-3′,5′), 6.89 (1H, s, H-3), 6.51 (1H, d, *J* = 1.8 Hz, H-8), 6.20 (1H, d, H-3, *J* = 2.4 Hz, H-6); ^13^CNMR(DMSO-*d*_*6*_, 150 M Hz) *δ*: 181.8 (C-4), 164.2 (C-7), 163.3 (C-2), 162.3 (C-4′), 161.4(C-5), 157.3 (C-9), 128.3 (C-2′,6′), 122.8 (C-1′), 114.5 (C-3′,5′), 103.8 (C-10), 103.5 (C-3), 98.9 (C-6), 94.0 (C-8). Compound 1 was identified as acacetin by comparison of its physical and spectral data with the literature [14,15].

### HPLC method validation

#### Calibration curve, LOD and LOQ

The calibration curve for acacetin was as y = 43237 x + 10969, R^2^ = 0.9999 (*P* < 0.001). The test range was 1.168-12.848 μg/mL. The LOD (S/N = 3) and LOQ (S/N = 10) was 0.29 μg/mL and 1.17 μg/mL, respectively.

#### Stability test

The RSD of peak areas at different times were less than 1.13%, indicating that the sample was stable for at least 24 h.

#### Precision test

The RSD value of intra-day and inter-day precision was 0.11% and 1.64%, respectively, which suggested that the developed method was precise enough for determining acacetin in *Z. clinopodioides*.

#### Accuracy test

The recoveries of acacetin were 97.33-103.92%, which indicated the developed method was suitable for determination of acacetin from *Z. clinopodioides* (Table 1).

**Table 1:** **Accuracy of the HPLC method for the determination of acacetin**

**Original acacetin (mg)**	**Spiked acacetin (mg)**	**Found acacetin (mg)**	**Recovery (%)**
0.02386	0	0.02386	
0.02257	0.01168	0.03471	103.92
0.02235	0.01168	0.03425	101.88
0.02226	0.01168	0.03430	103.10
0.02213	0.02336	0.04566	100.72
0.02195	0.02336	0.04488	98.14
0.02186	0.02336	0.04460	97.33
0.02252	0.03504	0.05735	99.40
0.02253	0.03504	0.05827	102.01
0.02222	0.03504	0.05773	101.35

### Quantitative analysis

**Figure 1:**
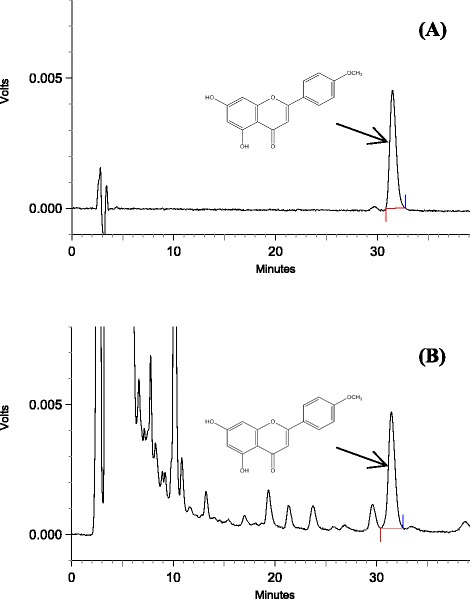
**HPLC chromatograms of the aerial part of** ***Z. Clinopodioides*** **. (A)** Acacetin. **(B)** Extract of sample.

Figure 1A shows an HPLC chromatogram for Acacetin, Figure 1B shows a chromatogram of extract of sample. The results of the quantitative analysis of three batches of *Z. clinopodioides* are shown in Table 2. No significant differences of acacetin content in *Z. clinopodioides* were found from one batch to another (ranging from 45.50 to 47.41 μg/g) (Table 2).

**Table 2:** **Content of acacetin in the aerial portion of** ***Z. clinopodioides*** **from different locations in Xinjiang (n = 3)**

**Species**	**Location**	**Acacetin (μg/g)**
*Z. clinopodioides*	AO^a^, South Mountain, Urumqi	45.60 ± 0.04
*Z. clinopodioides*	CT^b^, South Mountain, Urumqi	45.50 ± 0.10
*Z. clinopodioides*	Altai Mountain, Alaty	47.41 ± 0.18

^a^AO: Astronomical observatory.

^b^CT: Chrysanthemum terrace.

### Cell viability result

The cardiomyocyte viability was greater than 50% when subjected to an acacetin dose less than 12.5 μg/mL (Table 3).

**Table 3:** **Effects of different acacetin concentrations on cardiomyocyte viability**

**Concentration (μg/mL)**	**3.175**	**6.25**	**12.5**	**25.0**	**50**	**100**	**200**
Viability (%)^1^	54.78	52.37	52.93	47.76	45.48	43.11	44.49

^1^The results reported are the average of three experiments.

### Effects of acacetin on neonatal rat cardiomyocytes

The MDA content was significantly increased from 0.14 ± 21.34 to 30.72 ± 1.40 nmol/Lafter myocardial cell injury (Table 4). Compared with Group II (30.72 ± 1.40 nmol/L), the MDA content of Group III, Group VI, and Group V were reduced to 4.00 ± 2.91 nmol/L (*P* < 0.001), 10.64 ± 3.54 nmol/L (*P* < 0.001) and 15.45 ± 14.62 nmol/L (*P* = 0.033), respectively. These results confirmed that as the treatment concentration of acacetin increased, the MDA content in cardiomyocytes decreased. At 25 and 10 μg/mL acacetin, a significant reduction was observed (*P* < 0.001 and *P* < 0.001, respectively) (Table 4).

**Table 4:** **The effect of acacetin on the MDA content in neonatal rat cardiomyocytes subjected to hypoxia/reoxygenation injury**

**Groups**	**Acacetin (μg/mL)**	**MDA (nmol/L)** ^**1**^
I	—	0.14 ± 1.34
II	—	30.72 ± 1.40
III	25	4.00 ± 2.91^**^
IV	10	10.64 ± 3.54^**^
V	5	15.45 ± 14.62^*^

^1^Results are expressed as the mean ± S.D (n = 6).

^*^ *P* < 0.05, significant difference *vs.* group II.

^**^ *P* < 0.01 significant difference *vs.* group II.

## Discussion

Flavonoids are polyphenol compounds, which are widely distributed in a variety of plants and have many pharmacological activities associated with cardiovascular protection such as antioxidation [16], anti-inflammatory, blood vessel expansion, arrhythmia inhibition, and antiplatelet aggregation [17]. Some flavonoids also have antitumor activities [18,19].

Acacetin exists in plants of asteraceae [20–22], and violaceae [23], but was rarely identified in lamiaceae. Acacetin is an atrium-selective agent that prolongs the atrial refractory period without prolonging the corrected QT interval and effectively prevents atrial fibrillation in anesthetized dogs after intraduodenal administration. These results indicate that oral acacetin might be a promising atrium-selective agent for the treatment of AF [24]. However, the antioxidant activity of acacetin has not been thoroughly investigated.

In cardiomyocyte injury induced by hypoxia/reoxygenation, which is similar to heart ischemia-reperfusion injury *in vitro*, free radical injury was involved [25]. After myocardial ischemia-reperfusion, the body produces oxygen free radicals (OFR), and OFR-mediated cell membranes and subcellular membrane lipid peroxidation (LPO), while MDA is the LPO reaction product induced by OFR attacking the biomembrane. The amount of MDA reflects the degree of LPO, and is usually used to evaluate the degree of exposure to OFR [26]. In this study, after subjecting the cardiomyocytes to hypoxia/reoxygenation, the content of MDA in the medium increased significantly. Treatment with acacetin prevented the increase in MDA content, hence improving the antioxidant capacity of the myocardial cells.

## Conclusions

Acacetin protects neonatal cardiomyocytes from the damage induced by hypoxia/reoxygenation stress through reduction of lipid peroxidation and enhancement of the antioxidant activity.

## Abbreviations

HPLC: High-performance liquid chromatography
TLC: Thin layer chromatography
LOD: Limit of quantification
LOQ: Limit of quantification
RSD: Relative standard deviation
MTT: Methyl thiazolyl tetrazolium
DMEM: Dulbecco’s modified eagle’s medium: MDA, Malondialdehyde
CHCl_3_: Trichloromethane
EtOAc: Ethyl acetate
MeOH: Methanol
OFR: Oxygen free radicals
LPO: Lipid peroxidation
TBA: Thiobarbituric acid
AF: Atrial fibrillation
